# Allelic imbalance of somatic mutations in cancer genomes and transcriptomes

**DOI:** 10.1038/s41598-017-01966-z

**Published:** 2017-05-10

**Authors:** Je-Keun Rhee, Sejoon Lee, Woong-Yang Park, Young-Ho Kim, Tae-Min Kim

**Affiliations:** 1 Cancer Research Institute, College of Medicine, The Catholic University of Korea, 222 Banpo-daero, Seocho-gu, Seoul 06591 Republic of Korea; 2 Samsung Genome Institute, Samsung Medical Center, Seoul, Republic of Korea; 3 Translational Epidemiology Research Branch, Research Institute, National Cancer Center, Goyang, Republic of Korea; 4 Departments of Medical Informatics, College of Medicine, The Catholic University of Korea, 222 Banpo-daero, Seocho-gu, Seoul 06591 Republic of Korea

## Abstract

Somatic mutations in cancer genomes often show allelic imbalance (AI) of mutation abundance between the genome and transcriptome, but there is not yet a systematic understanding of AI. In this study, we performed large-scale DNA and RNA AI analyses of >100,000 somatic mutations in >2,000 cancer specimens across five tumor types using the exome and transcriptome sequencing data of the Cancer Genome Atlas consortium. First, AI analysis of nonsense mutations and frameshift indels revealed that nonsense-mediated decay is typical in cancer genomes, and we identified the relationship between the extent of AI and the location of mutations in addition to the well-recognized 50-nt rules. Second, the AI with splice site mutations may reflect the extent of intron retention and is frequently observed in known tumor suppressor genes. For missense mutations, we observed that mutations frequently subject to AI are enriched to genes related to cancer, especially those of apoptosis and the extracellular matrix, and C:G > A:T transversions. Our results suggest that mutations in known cancer-related genes and their transcripts are subjected to different levels of transcriptional or posttranscriptional regulation compared to wildtype alleles and may add an additional regulatory layer to the functions of cancer-relevant genes.

## Introduction

Cancer is a genomic disorder associated with various types of somatic alterations from small-scale single-nucleotide variations or substitutions (SNV) and short insertions/deletions (indels) to chromosome-scale copy number alterations and rearrangements^1, 2^. Advances in high-throughput genome sequencing technology have facilitated the sequencing of hundreds of thousands of cancer genomes, resulting in the identification of many cancer-related somatic mutations^3, 4^. SNVs and indels comprise a major fraction of somatic alterations in cancer genomes, especially those profiled by recent sequencing efforts. Their functional consequences have been inferred based on the amino acid alterations resulting from nucleotide-level changes. For example, truncating mutations including nonsense SNVs and frameshift indels as well as those occurring at splice sites are considered loss-of-function events, while synonymous or silent SNVs are considered functionally neutral. In the case of nonsynonymous or missense SNVs, a number of hotspot mutations with clinical relevance have been identified (i.e., *KRAS* p.G12D and *BRAF* p.V600E)^5, 6^. A number of methods have also been proposed to infer the potential functional consequences of missense mutations (SIFT, PolyPhen2, MutatorAssessor, etc.)^7–9^. However, it is a daunting task to identify the functional consequences of the missense mutations that comprise a major fraction of somatic alterations in cancer genomes.

Although the impact of the somatic mutation on the gene expression has been poorly understood, it is supposed that the somatic mutation may influence on the stability of mutant transcripts^10, 11^. The comparison of allelic frequencies of somatic mutations at DNA and RNA levels as measured from genome and transcriptome sequencing, respectively, can be used to assess the potential *cis*-impact of somatic mutations at the transcript level. The over- or under-representation of RNA transcripts harboring mutant alleles over wildtype alleles may be observed against the DNA-level mutant *vs*. wildtype allelic ratio, and this may indicate the presence of genetic mechanisms that are in favor of or against the mutant transcripts at the transcription or post-transcription level. It is well known that RNA transcripts with premature termination codons (PTC) are subject to nonsense-mediated decay (NMD), in which the transcripts with PTC will be degraded by the RNA surveillance mechanism^12^. The preference of NMD can be determined by the PTC position. If the PTC is located within 50 nt from the rightmost exon-exon junction or located at the last exon, the transcript may escape from NMD (50-nt rule)^12, 13^. NMD associated with germline variants has been recently demonstrated in large-scale paired transcriptome-genome sequencing data^14^. The roles of NMD in carcinogenesis have been proposed previously^15^, and its prevalence in cancer genomes has been recently investigated, focusing on ~3,000 nonsense mutations^16^. In addition, it has been proposed that non-mutated variants may be subject to NMD, suggesting that NMD is more prevalent than previously expected especially in cancer^17–19^. In addition, mutant-biased transcriptional up-regulation was observed for some missense SNVs such as those on *EGFR* and *KRAS* ^20, 21^. This observation suggests that some cancer-related somatic mutations are subjected to different levels of transcription or post-transcriptional regulation; however, the systematic understanding of such allelic imbalance (AI) between the genome and transcriptome of somatic mutations in cancer genomes is still lacking.

In this study, we performed a large-scale AI analysis of the somatic mutations in cancer genomes (hereafter, AI is defined as the mutant *vs*. wildtype allelic imbalance as measured between DNA and RNA levels, where the DNA- and RNA-level mutation abundances are measured from whole-exome and transcriptome sequencing data). The main objective of our study was to investigate the impact of major types of somatic mutations in terms of AI reflecting their potential transcriptional or post-transcriptional regulatory mechanisms. For the study of AI, we collected sequencing data from the large-scale cancer genome database of the Cancer Genome Atlas (TCGA) consortium. Five major human tumor types (breast, head and neck, kidney, lung, and stomach) of >2,000 patients were used in this study. We collected a large number of somatic mutations and performed AI analyses with respect to the mutation categories of missense, silent, nonsense, and splice site SNVs along with frameshift and inframe indels. Our systematic analysis revealed that the somatically mutated genes often showed AI of transcription, and the preference for allelic expression was diverse across the mutation types.

## Results

### Dataset and somatic mutations

For AI analyses, we first collected a total of 368,921 somatic mutations (332,225 SNVs and 36,696 indels) from 2,243 tumor specimens across five tumor types (858 breast cancers/BRCA; 486 head and neck cancers/HNSC; 161 kidney renal clear cell carcinomas/KIRC; 465 lung adenocarcinomas/LUAD; 273 stomach adenocarcinomas/STAD) from the TCGA portal (https://tcga-data.nci.nih.gov/). We categorized the somatic mutations into four SNV classes (missense, nonsense, splice site, and silent SNV) and two indel classes (frameshift and inframe), respectively. The mutant and wildtype allele counts of individual somatic mutations at DNA and RNA levels were also obtained from TCGA tumor BAM files as whole-exome sequencing (WES) and transcriptome sequencing (RNA-seq), respectively. We further selected the mutations supported by no less than 10 sequencing reads both in WES and RNA-seq datasets to obtain initial candidates of AI analyses. Finally, we selected 103,661 mutations (99,235 SNPs and 4,426 indels) observed in 13,687 genes and 2,112 tumor specimens. The preprocessing procedure is illustrated in Supplementary Figure S1. The somatic mutations used for AI analyses is summarized in Table 1.

**Table 1:** Number of mutations across five tumor types.

	BRCA	HNSC	KIRC	LUAD	STAD
**SNV #**					
Nonsense	1,129 (5.2%)	1,704 (5.3%)	133 (3.3%)	1,829 (5.2%)	563 (3.8%)
Missense	14,066 (65.4%)	20,308 (63.4%)	1,896 (46.9%)	23,091 (65.0%)	9,257 (61.9%)
Splice_Site	74 (0.3%)	117 (0.4%)	8 (0.2%)	147 (0.4%)	44 (0.3%)
Silent	4,281 (19.9%)	7,968 (24.9%)	728 (18.0%)	8,311 (23.4%)	3,581 (23.9%)
**INDEL #**					
Frameshift	800 (3.7%)	733 (2.3%)	540 (13.4%)	787 (2.2%)	581 (3.9%)
Inframe	187 (0.9%)	246 (0.8%)	97 (2.4%)	280 (0.8%)	175 (1.2%)

### AI of somatic mutations in WES and RNA-seq

**Figure 1:**
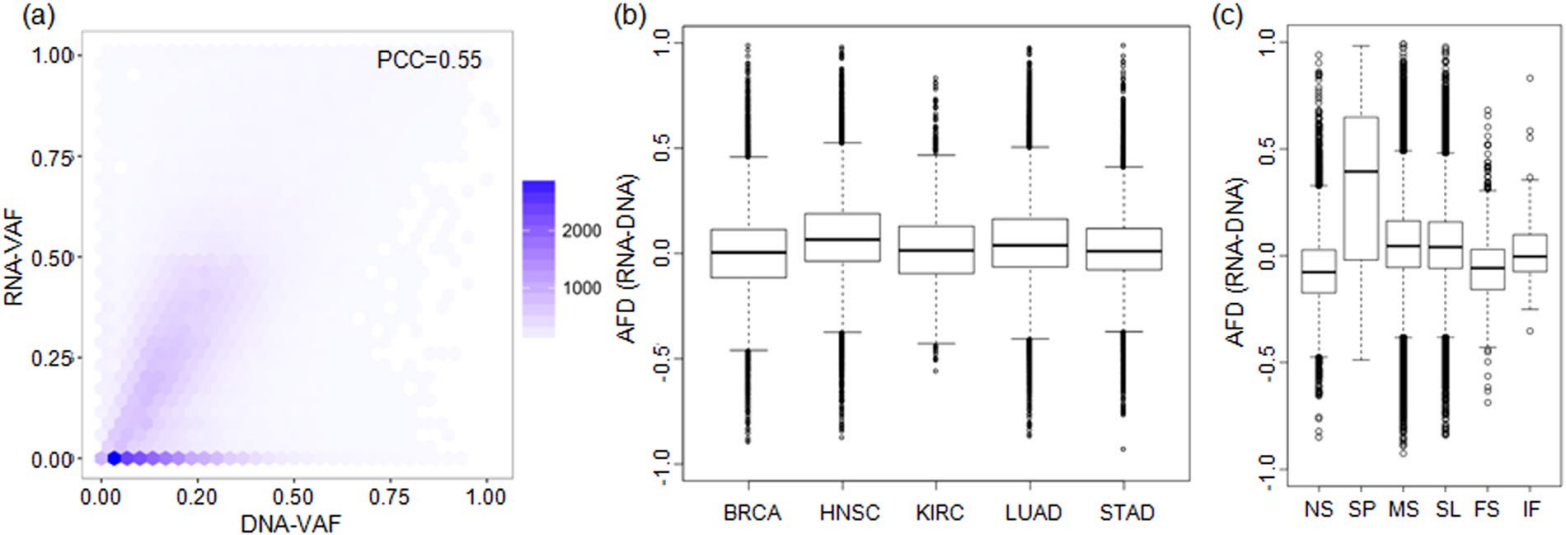
VAF and somatic mutations. (**a**) VAF of individual mutations observed in WES and RNA-seq are shown in *x*- and *y*-axis, respectively. The number of points or mutations is represented by the color density. PCC is Pearson correlation coefficient (*P* value was not shown because it was negligible). (**b**,**c**) Boxplots of AFD are shown for five tumor types (**b**) and for six mutation categories (**c**). NS, nonsense mutations; SP, splice site mutations; MS, missense mutations; SL, silent mutations; FS, frameshift indels; IF, inframe indels.

For each somatic mutation, we calculated the variant allele frequency (VAF) of the DNA and RNA from the mutant and wildtype allele counts of WES and RNA-seq, respectively. The distributions of RNA-VAF and DNA-VAF of all the mutations across the five tumor types are shown in Fig. 1(a) and Supplementary Figure S2. The distribution of allelic fraction difference (AFD) (RNA-VAF minus DNA-VAF) for individual tumor types is shown in Fig. 1(b). In these plots, the majority of mutations showed comparable DNA- and RNA-VAF, consistent with a report that a majority of mutations are transcribed according to DNA frequencies^22^. When AFD was plotted with respect to the mutation categories, some mutations showed AI of the gene expression, as demonstrated by a bias between the DNA- and RNA-VAFs (Fig. 1(c)). The biases of AFD or AI are primarily notable for the nonsense SNVs and frameshift indels toward negative AFD (RNA-VAF < DNA-VAF) and for the splice site mutations toward positive AFD (RNA-VAF > DNA-VAF), respectively.

### NMD and 50-nt rule

**Figure 2:**
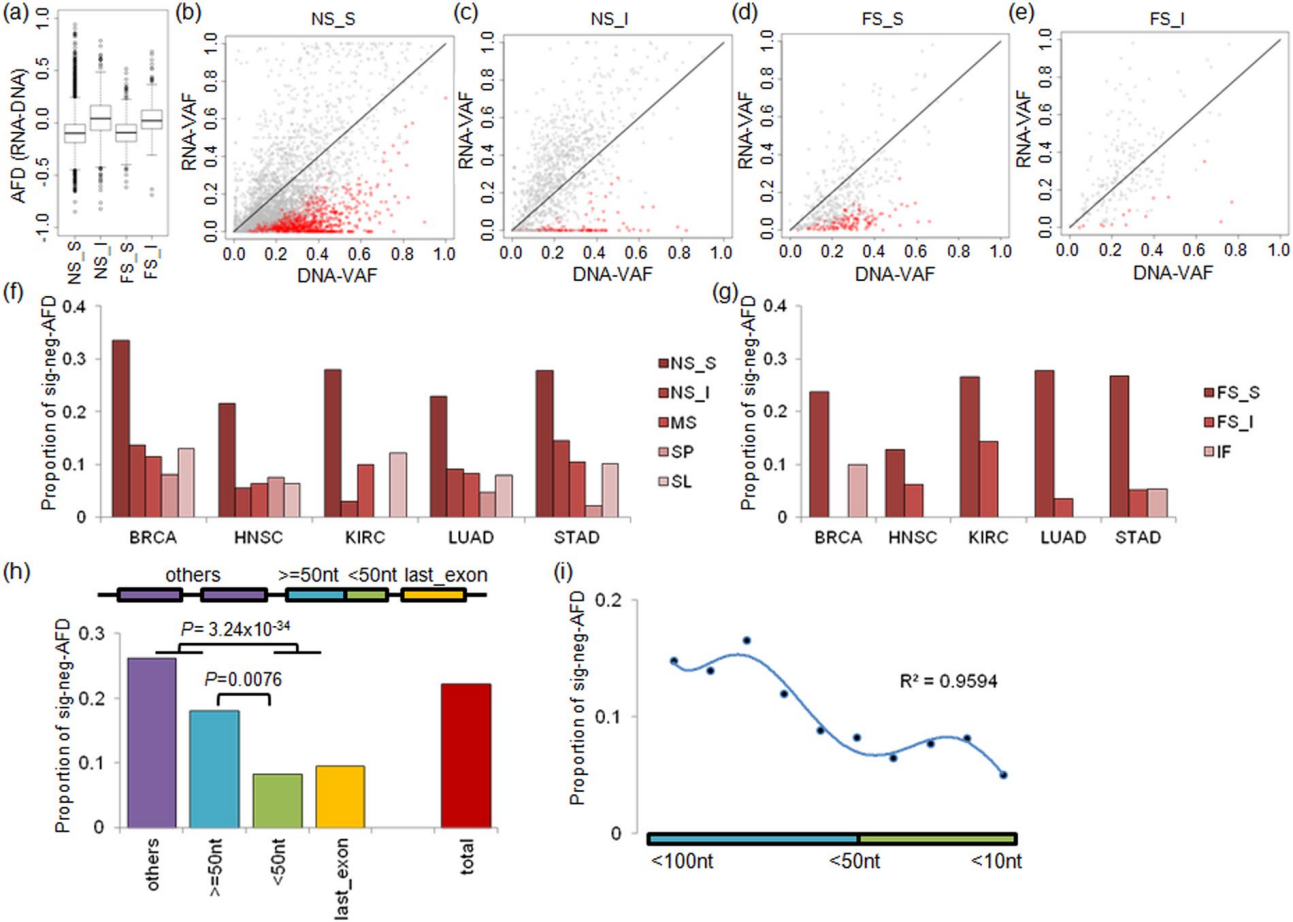
NMD and the 50-nt rule. (**a**) The boxplot shows AFD of NMD-sensitive and -insensitive variants. The NMD-sensitive and -insensitive variants are classified according to the 50-nt rule. NS_S, NMD-sensitive nonsense mutations; NS_I, NMD-insensitive nonsense mutations; FS_S, NMD-sensitive frameshift indels; FS_I, NMD-insensitive frameshift indels. (**b–e**) Scatterplots show the DNA-VAF and RNA-VAF observed in NS_S (**b**), NS_I (**d**), FS_S (**e**), FS_I (**f**). Red indicates sig-neg-AFD mutations. (**f**) Barplots are shown for the sig-neg-AFD ratio of SNVs in individual tumor types. The sig-neg-AFD ratio was calculated as the number of sig-neg-AFD mutations divided by the total number of the somatic mutations in each tumor type. (**g**) Similarly shown for indels. (**h**) The sig-neg-AFD ratios shown for the last exonic region (yellow), >= 50 nt and <50 nt in the penultimate exon (blue and green, respectively) and the other exonic regions (purple). The sig-neg-AFD ratio for all the exonic region is shown at the rightmost bar (red). (**i**) The sig-neg-AFDs ratios are shown with respect to the distance of PTC from the last exon-exon junction at the penultimate exon. The *x*-axis is represented as a cumulative scale, that is <10 nt is 0 to 10 nt, <50 nt was 0 to 50 nt, and <100 nt is 0 to 100 nt. The point is marked on every 10 nts.

The preference of negative AFD for nonsense SNVs and frameshift indels is consistent with a known biological phenomenon of NMD where the transcripts containing those truncating mutations are relatively deficit compared to those with wildtype alleles. According to the 50-nt rule^12, 13^, we further categorized the nonsense SNVs and frameshift indels into NMD-sensitive mutations (i.e., those with PTC located more than 50 nucleotides upstream of the 3′ most exon-exon junction) and NMD-insensitive mutations. The extent of AI was more apparent for NMD-sensitive mutations than NMD-insensitive mutations both for the nonsense SNVs and frameshift indels, suggesting that the 50-nt rule for NMD-sensitivity is true for cancer genomes (Fig. 2(a)). The RNA-VAFs were significantly lower than DNA-VAFs only for the NMD-sensitive nonsense SNVs and frameshift indels (*P* value 1.14 × 10^−188^ and 1.35 × 10^−34^, respectively; one-tailed paired *t*-test). No significant difference was observed between the DNA-VAF and RNA-VAF for NMD-insensitive nonsense SNVs, frameshift indels, or other variants including missense SNVs, silent SNVs, and inframe indels.

To obtain high-confidence NMD events, the differences in allelic proportions were evaluated by a Bayesian hypothesis testing frameworks identifying the individual mutations that are subject to NMD (sig-neg-AFD; RNA-VAF < DNA-VAF) as previously described by Rashid *et al*.^23^. As expected, the proportion of sig-neg-AFD mutant transcripts representing NMD events was significantly higher for the nonsense mutations than for other somatic mutation classes (Supplementary Figure S3; *P* value 5.28 × 10^−181^; Fisher’s exact test). The scatterplots of RNA-VAF and DNA-VAF for the NMD-sensitive and -insensitive nonsense SNVs and frameshift indels also show the relative enrichment of sig-neg-AFD mutations in NMD-sensitive mutations compared to NMD-insensitive mutations (Fig. 2(b)–(e); red dots for sig-neg-AFD mutations). To further investigate mutations subject to NMD in known cancer-related genes, we obtained a list of 244 genes (116 oncogenes, 91 tumor suppressor genes or TSGs and 37 genes both annotated as oncogenes and TSGs) from the Cancer Gene census^24^ in the COSMIC database^25^. The sig-neg-AFD mutations were more enriched in TSGs compared to oncogenes, e.g., 54 and 16 sig-neg-AFD mutations were observed in 20 TSGs and 12 oncogenes, respectively. In addition, 30 sig-neg-AFD mutations were observed on 6 genes both annotated as oncogenes and TSGs while the majority of them (*n* = 23) were observed in *TP53*. Moreover, for SNVs, the significant enrichment of sig-neg-AFD mutations were observed in the NMD-sensitive nonsense SNVs compared to other classes including the NMD-insensitive nonsense SNVs across the five tumor types (Fig. 2(f); *P* value 1.96 × 10^−214^; Fisher’s exact test). Similarly, the abundance of sig-neg-AFD was significantly higher for NMD-sensitive frameshift indels compared to inframe indels and frameshift indels with no PTC or NMD-insensitive PTC across the tumor types examined (Fig. 2(g); *P* value 5.05 × 10^−13^; Fisher’s exact test). To further investigate the 50-nt rules, we compared the proportion of sig-neg-AFD in four entities according to the occurrence of PTC (i.e., >=50 nt and <50 nt in the penultimate exon, the last exon, and the others) (Fig. 2(h)). The proportion of the sig-neg-AFD was clearly demarcated at the 50 nt from the last exon-exon junction (*P* value < 0.008; Fisher’s exact test). In addition, the proportion of sig-neg-AFD measured in 10 bp-bins of the distant between PTC and the last exon junction further supports that the 50 nt rule is an important factor in NMD efficiency (Fig. 2(i)).

### NMD can be escaped by other factors

**Figure 3:**
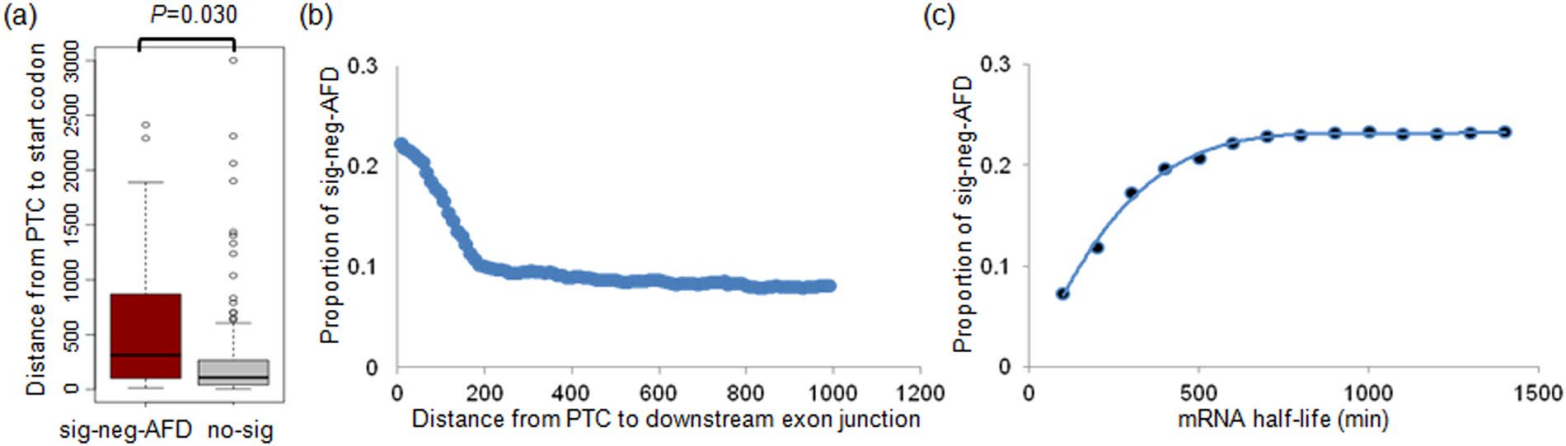
The relationship between the location of PTC and NMD. (**a**) The distance of PTC from start codons are shown for sig-neg-AFD mutations (red) and the other mutations (gray). (**b**) Scatterplot shows the distance of PTC to downstream exon junction. The *x*-axis is represented as a cumulative sum. The point is marked on every 10 nts. (**c**) The sig-neg-AFDs ratio are plotted against the mRNA half-life. The minutes on the x-axis are represented as a cumulative scale.

To identify additional features associated with NMD, we separately analyzed the proportion of sig-neg-AFDs in nonsense mutations at the first exon versus mutations elsewhere. The proportion in the first exon was significantly lower (Supplementary Figure S4; *P* value 1.66 × 10^−5^; Fisher’s exact test). The relatively small fraction of 12.10% (19 nonsense mutation) of nonsense SNVs in the first exons among sig-neg-AFD events suggests that the PTC in the first exons is less vulnerable to NMD, which is consistent with the idea that the transcript harboring PTC close to the start codon may be insensitive to NMD^26^. For nonsense mutations in the first exon, the distance from the start codon was investigated, and we found that sig-neg-AFD events were significantly distant from the start codon compared to others (*P* value 0.030; one-tailed t-test; Fig. 3(a)), suggesting that PTC close the start codons are relatively insensitive to NMD.

Next, as a potential factor related to the NMD sensitivity, we examined the distance between the PTC and downstream exon junction with the PTC at exons excluding the first, last, and penultimate exons. Figure 3(b) shows the sig-neg-AFD proportion and the length as a cumulative value. The proportion of sig-neg-AFD was sharply decreased at a distance of 200 nt. This suggests that PTC distant from the downstream exon-junction complex are less vulnerable to the NMD compared to those close to the downstream exon junction. Figure 3(c) show the relationships between mRNA half-life and sig-neg-AFD, where sig-neg-AFD is more frequently observed for genes with a long mRNA half-life. These observations further show that the relative location of PTC from the start codons and downstream exon junctions may be a fine-tuning factor in determining NMD in addition to the 50-nt rule.

### AI of splice site mutations

**Figure 4:**
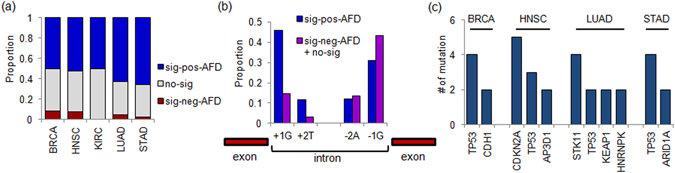
AI and splice site mutations. (**a**) Barplots show the relative proportion of sig-pos-AFD (blue), sig-neg-AFD (red), and the other mutations (gray) for splice site mutations across five tumor types. (**b**) The sig-pos-AFD ratio (blue) and the other ratio (purple) are shown at the canonical 5′ splice site (GT) and 3′ splice site (AG). (**c**) The frequency of the splice site mutations with the sig-pos-AFD are shown for genes with the ≥ 2 sig-pos-AFD events in the given tumor type.

Aberrant splicing may play a pathogenic role in a variety of human diseases including cancers^27, 28^. It has been shown that splice site mutations can produce abnormal splicing events in an allele-specific manner, including intron retention and exon skipping^29^. As shown Fig. 4(a), most of the splice site mutation-harboring alleles are relatively over-expressed compared to the wildtype alleles (0.50–0.66% of sig-pos-AFD across five tumor types), indicative of retention of 5′ or 3′ intronic mutant alleles in the transcripts. Given that the proportion of sig-pos-AFD of splice site mutations reflects the extent of splicing disruption, we further investigated the relationship between abundance of sig-pos-AFD and the position of mutations with respect to the splice site (5′ GT and 3′ AG) (Fig. 4(b)). AI was dominantly observed for mutations at intronic 5′ splice sites compared to those at 3′ sites (*P* value 2.19 × 10^−09^; Fisher’s exact test). No significant difference in the mutation spectra was observed with respect to the mutation abundance of sig-pos-AFD-vs.-others (Supplementary Figure S5; *P* value > 0.1; Fisher’s exact test). Among 223 sig-pos-AFD splice site mutations, 29 and 4 mutations were observed in 8 TSGs and 4 oncogenes, respectively. Sixteen mutations were found on 4 genes both annotated as oncogenes and TSGs including 13 mutations in *TP53* gene. Figure 4(c) lists the genes with more than one sig-pos-AFD event at splice site mutations across tumor types where known TSGs such as *TP53, CDKN2A*, *STK11*, and *ARID1A* frequently harbored sig-pos-AFD at splice site mutations. Although *HNRNPK* was not included in the cancer gene census as a TSG, it may be a haploinsufficient tumor suppressor in hematologic malignancies^30^. This finding suggests that the major targets of sig-pos-AFD at splice site mutations are putative TSGs leading to the aberrant splicing (i.e., intron retention) of them as one of potential inactivating mechanisms of TSGs^29^.

### AI of missense mutations

**Figure 5:**
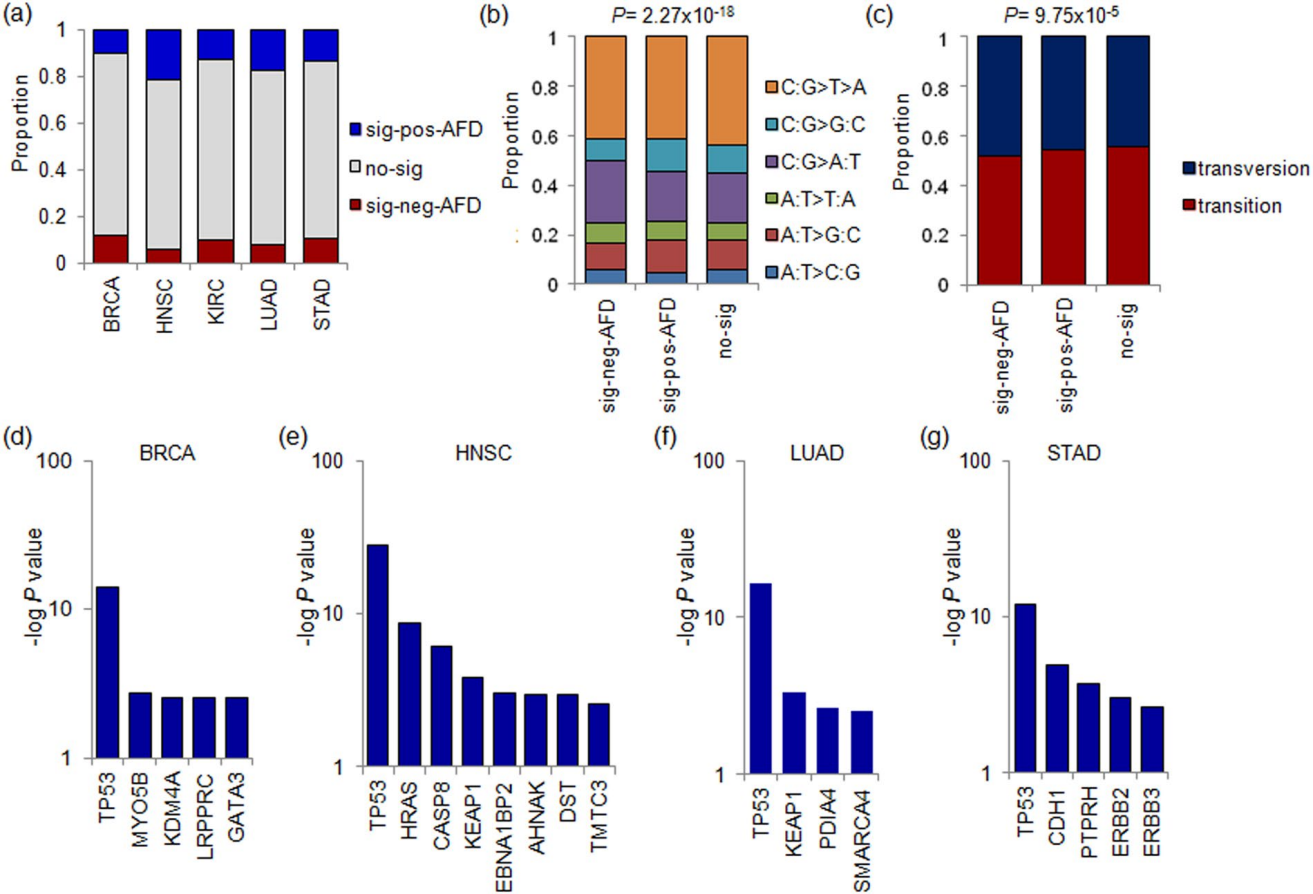
AI at missense mutations. (**a**) Barplots show the relative proportion of sig-pos-AFD (blue), sig-neg-AFD (red), and the other mutations (gray) for missense mutations across five tumor types. The proportion was calculated for events in copy number neutral segments. (**b**) The mutation spectra are shown for the sig-pos-AFD, sig-neg-AFD and the other mutations. *P* value was calculated by Chi-square test. (**c**) The proportion of the transversion and transition mutations are shown for sig-pos-AFD, sig-neg-AFD and the others. *P* value was calculated by Chi-square test. (**d**) Significance level of genes are shown for the enrichment of sig-pos-AFD missense mutations in BRCA. *Y*-axis is -log *P* value (Fisher’s exact test). Genes with -log *P* value > 2.5 are shown. Similarly shown for HNSC (**e**), LUAD (**f**) and STAD (**g**). KIRC has no genes to be plotted by the criterion.

Some cancer-related missense mutations such as those of *KRAS* show elevated transcript level compared to wildtype^21^. Across five tumor types, 11.1–20.4% and 6.4–11.4% of missense mutations were classified as sig-pos-AFD or sig-neg-AFD, respectively, suggesting that a substantial number of missense mutations are associated with AI (Supplementary Figure S6). To rule out the potential impact of copy number alterations on AI, we filtered the mutations in copy number neutral regions, observing proportions of sig-pos-AFD and sig-neg-AFD of 10.0–21.4% and 6.1–11.5%, respectively (Fig. 5(a)). The subsequent analyses were performed with copy number neutral missense mutations. First, we explored the mutation spectra of the sig-pos-AFD, sig-neg-AFD, and the other no-sig missense mutations (Fig. 5(b)). The genomic substitution landscapes were significantly different between the sig-pos-AFD and sig-neg-AFD sites (*P* value = 2.27 × 10^−18^; Chi-square test), showing an overrepresentation of C:G > A:T in sig-neg-AFD mutations (transversions) compared to other mutations. Consistently, when we compared the number of transitions and transversions for the missense mutations, the frequency of transitions at sig-pos-AFD was also significantly higher (*P* value = 9.75 × 10^−5^; Chi-square test; Fig. 5(c)).

To determine the suspected roles of missense mutation and the variant allele transcripts, we investigated genes harboring sig-pos-AFD mutations. Although the number of the TSGs and oncogenes with sig-pos-AFD were comparable (e.g., 323 mutations at 67 TSGs and 340 mutations at 76 oncogenes), we note that a substantial number of the genes with frequent sig-pos-AFD AI events are cancer-related genes. For example, Fig. 5(d)–(g) show genes with frequent sig-pos-AFD AI events in each tumor type and 9 of 18 genes listed in the Figures were noted as known cancer-related genes. Supplementary Figure S7 presents genes with frequent sig-pos-AFD across all cases examined. In our analysis, one of the most representative TSGs was*TP53* mutant alleles, which were found to be preferentially expressed in most tumor types, BRCA, HNSC, LUAD, and STAD (*P* values < 1.25 × 10^−12^ in each type; Fisher’s exact test), excluding KIRC, as also previously observed in another tumor^31^. Among the genes, *GATA3* was specifically founded in BRCA (*P* = 0.0028). *GATA3* is the most abundant transcription factor in luminal epithelial cells, and its potential role in breast epithelial carcinogenesis has been recently highlighted^32^. As a histone modifier and mitochondria-related autophagy inhibitor, the therapeutic potentials of *KDM4A* and *LRPPRC* have been noted^33, 34^. In addition to *TP53*, two TSGs, *CASP8* and *KEAP1*, frequently showed sig-pos-AFD (8.11 × 10^−07^ and 1.39 × 10^−04^, respectively) in HNSC. *CASP8* initiates programmed cell death as a well known TSG along with *KEAP1*, which is known to regulate oncogenic factor *NRF2* ^35^. *SMARCA4* (*P* = 2.78 × 10^−3^) and *KEAP1* (*P* = 4.93 × 10^−5^) were found to be significantly associated with sig-pos-AFD in LUAD. For STAD, a known TSG, *CDH1*, was also found to harbor significantly frequent sig-pos-AFD (*P* = 1.28 × 10^−5^), and it has been shown to have roles in multiple cancers including gastric cancer^36, 37^. These results suggest that the preferential expression of mutated alleles is frequent for known or potential tumor suppressor genes. Although we filtered out the mutations on copy number gained or lost regions, it is still possible that low frequent or subclonal copy number deletions that are frequently associated with TSGs may explain the frequent AI of TSGs. Further investigation is necessary to determine whether there exists a transcriptional or post-transcriptional mechanism in favor of transcripts harboring mutations of TSGs. Although the sig-pos-AFD enriched genes are largely tumor suppressive, *HRAS* and *ERBB2*, which are as well-known canonical oncogenes, are observed to be significantly enriched with sig-pos-AFD missense mutations (*P* value of 2.37 × 10^−09^ in HNSC and 9.03 × 10^−4^ in STAD, respectively; Fig. 5).

To further examine the functional effects of the statistically significant genes, we carried out preranked version of Gene Set Enrichment Analysis (GSEA)^38^. GSEA results showed that the genes with AI, especially sig-pos-AFD, were mostly enriched in the functional genesets representing apoptosis or cell differentiation (Supplementary Tables S1–S6). The molecular functions of the enriched genesets were largely similar across the tumor tissues except for KIRC. We also investigated the enriched functions of genes harboring sig-neg-AFD missense mutations. Supplementary Figure S8 shows genes with a significant number of sig-neg-AFD in each tumor type, and Supplementary Figure S9 presents aggregates of sig-neg-AFD genes across all tumor types examined. Supplementary Tables S7–S12 show that the frequently enriched molecular functions are those of collagen-related functions or ECM (extracellular matrix). ECM is one of the major contributors to tumor progression, and collagen is a major component of the ECM. Further analysis is required to understand the biological significance of the diminished mutant transcript level for collagen- or ECM-related genes.

## Discussion

The plethora of cancer genome and transcriptome sequencing data in the public domain has opened opportunities to perform large-scale AI studies analyzing the mutant allele abundance between DNA and RNA. In this study, we assessed the extent of AI of cancer-related somatic mutations across multiple types of cancers to gain additional insights into the potential *cis*-transcriptional regulatory effects of cancer mutations. A statistical model was employed to evaluate the imbalance of the number of reads with mutant and normal alleles in the exome (DNA) and transcriptome (RNA) sequencing data from the same patient. A major advantage of this approach is that the two alleles are measured from identical samples, and it can minimize the potential impact of technical issues such as tumor purity and copy numbers. Without assuming the copy number states of somatic mutations, we directly compared the proportion of variants allele measured by the sequence reads between DNA-based and RNA-based sequencing datasets.

First, we demonstrate that AI analysis with respect to different types of somatic mutations may be used to evaluate previously recognized biological phenomena. For example, 50-nt rules and exon-junction complex (EJC) models are well-recognized for NMD where the EJC proteins bind at least 50 nt upstream of the last exon junctions and will remain bound during protein translation and trigger the degradation of transcripts with PTC^12, 13^. Our study demonstrates that the 50-nt rules of the EJC model are also true for cancer genomes, and NMD may serve as an important mechanism to inactivate genes harboring truncating mutations, especially TSGs. In addition, we observed that PTC present outside the penultimate exon (e.g., the first exon or those between the first exons and the penultimate exon) might be associated with NMD according to distance from the start codon or from the downstream exon junctions. These findings enrich our insights into the relationship between the location of PTC and NMD in addition to the well-recognized 50-nt rules. For splice site mutations, those at the first intronic nucleotide (+1G) are the most enriched with sig-pos-AFD events, suggesting that these splice site mutations are more likely to experience intron retention. Our results imply that the G nucleotide at the 5′-end of the intron may be critical in the splicing function, and the mutations of that position will convert the sites into weaker splice sites^39^. Along with the splice site mutations with AI that are enriched in TSGs, we also observed that allele-specific expression at loci with missense mutations are common in known cancer-related genes. Although the genetic mechanisms that are responsible for the AI in missense mutations should be further investigated, known cancer-related genes with mutations may be subject to different transcriptional or posttranscriptional regulation leading to AI.

In this study, we set the minimum sequencing depth of mutations for AI analysis to be 10. Thus, among the mutations found in WES by conventional mutation calling algorithm^40, 41^, those supported by no less than 10 DNA (WES) and 10 RNA (RNA-seq) reads were subsequently analyzed for AI. Since WES and RNA-seq may give uneven coverage across the loci largely due to the extent of DNA capture and the level of transcription, respectively, this may affect the confidence of our AI analysis that is based on a Bayesian statistical test. Thus, we performed additional experiments to investigate the impact of minimum sequencing depth cutoff differentially set for WES and RNA-seq. Supplementary Figure S10 shows the proportion of sig-pos-AFD, sig-neg-AFD and no-sig events identified with the cutoff of 0–50 minimum WES reads and 0–50 minimum RNA-seq reads for three types of mutations. The figure shows that the proportion of the AI events are relatively constant with respect to the minimum DNA depth levels, but the proportions are substantially different with the minimum RNA depth level. Of note, increased RNA depth level captured more events corresponding to the NMD-suggesting sig-neg-AFD events for nonsense mutations and intron retention-suggesting sig-pos-AFD events for splice site mutations. The finding suggests that a sufficient RNA sequencing depth may be required for the robust identification of AI analysis, which requires further investigation for the optimal setting for the DNA and RNA sequencing depth for AI analyses.

Since we focused on somatic mutations in coding regions as covered by WES, the AI in promoter or other non-coding regions was not evaluated. As previous studies have proposed that a substantial number of genes may have allele-specific expression with *cis*-regulatory variation^42, 43^, whole-genome scale AI analysis including noncoding regions will be required to elucidate the allele-specific expression beyond the coding regions. Therefore, further investigation combining other genomic and epigenomic factors including DNA methylation or three-dimensional DNA conformation will be helpful to understand the complete processes for AI and to shed light on clinical applications.

## Methods

### Datasets

We selected the five tumor types of breast cancer (BRCA)^44^, head and neck carcinoma (HNSC)^45^, kidney renal clear cell carcinoma (KIRC)^46^, lung adenocarcinoma (LUAD)^47^, and stomach adenocarcinoma (STAD)^48^ from the TCGA consortium for AI analyses. First, we obtained somatic mutations from MAF (mutation annotation format) files generated by TCGA consortium and curated by TCGA Analysis Working Groups (https://gdc-portal.nci.nih.gov/). The data for BRCA, HNSC, LUAD, STAD was generated by Illumina Genome Analyzer IIx. But, for KIRC, the data was generated by Illumina Genome Analyzer IIx, Illumina HiSeq, and ABI SOLID systems. To obtain allele counts of individual somatic mutations at DNA and RNA levels, we downloaded TCGA tumor BAM files as WES and RNA-seq in dbGaP (http://www.ncbi.nlm.nih.gov/gap)^49^ with controlled access authorization. The sequencing data have been recently moved to Genomics Data Commons Data Portal (https://gdc-portal.nci.nih.gov/). The platforms used to generate raw sequencing data were Illumina GenomeAnalyzerII or HiSeq. Mutant or wildtype allele count or the number of sequencing reads supporting the mutations was obtained using Samtools with mpileup option after removing the sequencing reads with low mapping quality with the option of mpileup -q 1^50^. The mutations covered by no less than 10 WES and 10 RNA-seq reads were used for the subsequent AI analyses. The annotation of somatic mutations in MAF files were re-examined by ANNOVAR^51^. We discarded mutations with inconsistent annotations between the ANNOVAR- and original MAF-based annotations and also discarded mutations in genes harboring multiple mutations. The hypermutated cases (i.e., those with more than 10 mutations/Mb) were also excluded from the AI analyses. The NMD-sensitive/-insensitive nonsense mutations were discriminated as those >50 bp and <50 bp with respect to the most 3′ exon-exon junction. The same discrimination was made for indels using SIFT-annotated PTC location^52^. The overall procedures and filtering schemes are illustrated in Supplementary Figure S1.

### AI analyses

VAF represents the fraction of the number of reads with variants or mutant alleles in the total number of reads at the mutated sites. For individual mutations, DNA- and RNA-VAFs were obtained from WES and RNA-seq, respectively. The AFD is defined as the RNA-VAF minus DNA-VAF in the range of [−1,1]. We further defined the mutations with positive AFD (pos-AFD; RNA-VAF > DNA-VAF) as those with the mutant alleles preferentially expressed compared to wildtype alleles, and *vice versa* for the mutations with negative AFD (neg-AFD; RNA-VAF < DNA-VAF). The significance of the difference in DNA-VAF and RNA-VAF was estimated by a Bayesian hypothesis testing framework as previously described by Rashids *et al*.^23^. The threshold of significance for the allelic differential expression was determined as Bayes factor > 10, as previously recommended. Finally, we defined sig-pos-AFD and sig-neg-AFG mutations as those of pos-AFD and neg-AFD with Bayes factor > 10, respectively. To select the copy number neutral mutations, we obtained the copy number segmentation files of Affymetrix SNP6.0 filtered for the germline copy number variations. By intersecting the copy number segments and somatic mutations of the same patients, the mutations that belonged to segments with log ratios > −0.15 and <0.15 were considered copy number neutral mutations and used for the AI analyses. For investigation of factors related to NMD, mRNA half-life information was acquired from Liderboom’s study^16^.

The functional analysis of genes with sig-pos-AFD and sig-neg-AFD missense mutations was carried out using preranked version of GSEA. A joint set of MSigDB c2cp (curated canonical pathways) and c5 (Gene Ontology) was used as the functional gene set for the analysis. The input data, which was a ranked gene list, was compiled using the negative log *P* value obtained from a one-tailed Fisher’s exact test.

## Electronic supplementary material

Supplementary Figures

Supplementary Tables
