# Supplementary material for: Allelic imbalance of somatic mutations in cancer genomes and transcriptomes
Source: Sci Rep. 2017 May 10;7:1653. doi: 10.1038/s41598-017-01966-z (PMC5431982; doi:10.1038/s41598-017-01966-z)
Supplement: Supplementary file 1 — Supplementary Figures [file 41598_2017_1966_MOESM1_ESM.pdf]

Allelic imbalance of somatic mutations in cancer genomes and transcriptomes

Je-Keun Rhee[1], Sejoon Lee[2], Woong-Yang Park[2], Young-Ho Kim[3], and Tae-Min Kim[1,4,*]

[1] Cancer Research Institute, College of Medicine, The Catholic University of Korea, 222 Banpo-daero, Seocho-gu, Seoul 06591, Republic of Korea
[2] Samsung Genome Institute, Samsung Medical Center, Seoul, Republic of Korea
[3] Translational Epidemiology Research Branch, Research Institute, National Cancer Center, Goyang, Republic of Korea
[4] Departments of Medical Informatics, College of Medicine, The Catholic University of Korea, 222 Banpo-daero, Seocho-gu, Seoul 06591, Republic of Korea

* To whom correspondence should be addressed. Tel: +82-2-2258-7384; Email: tmkim@catholic.ac.kr

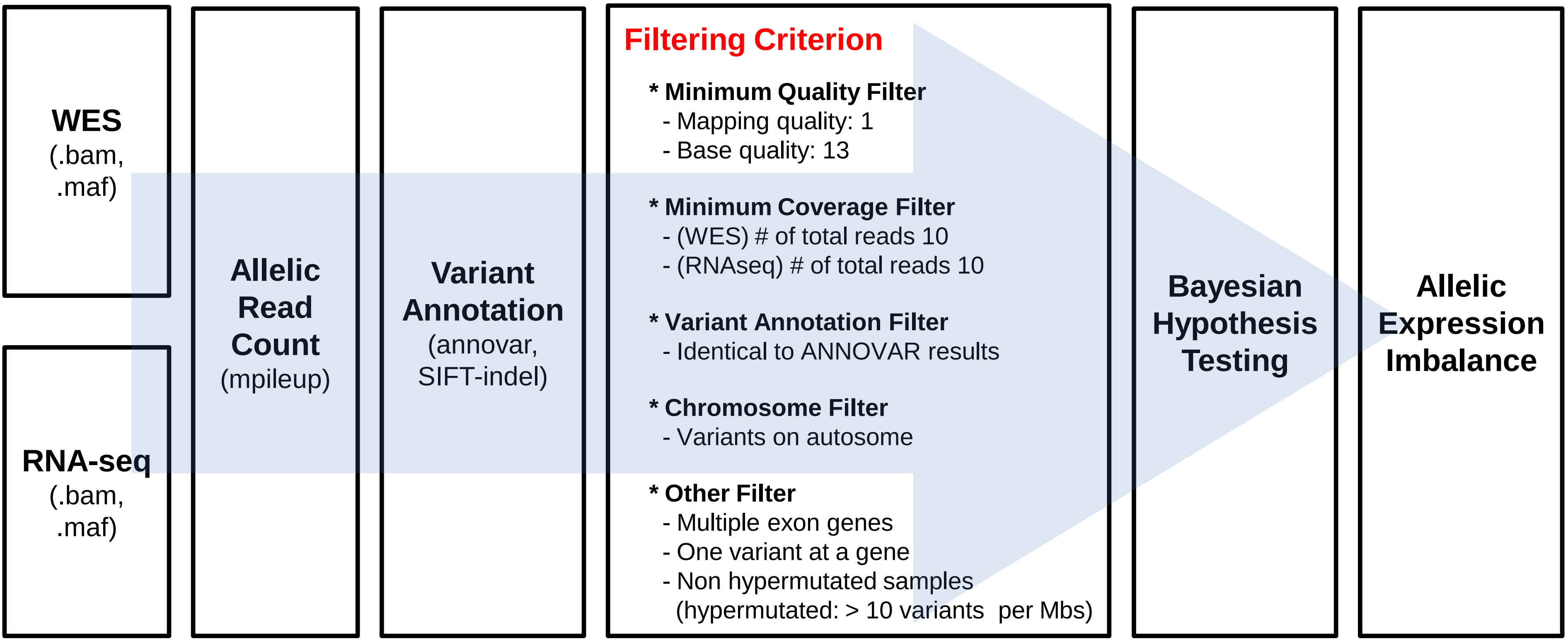

Fig. S1. Overall procedures and filtering criterion

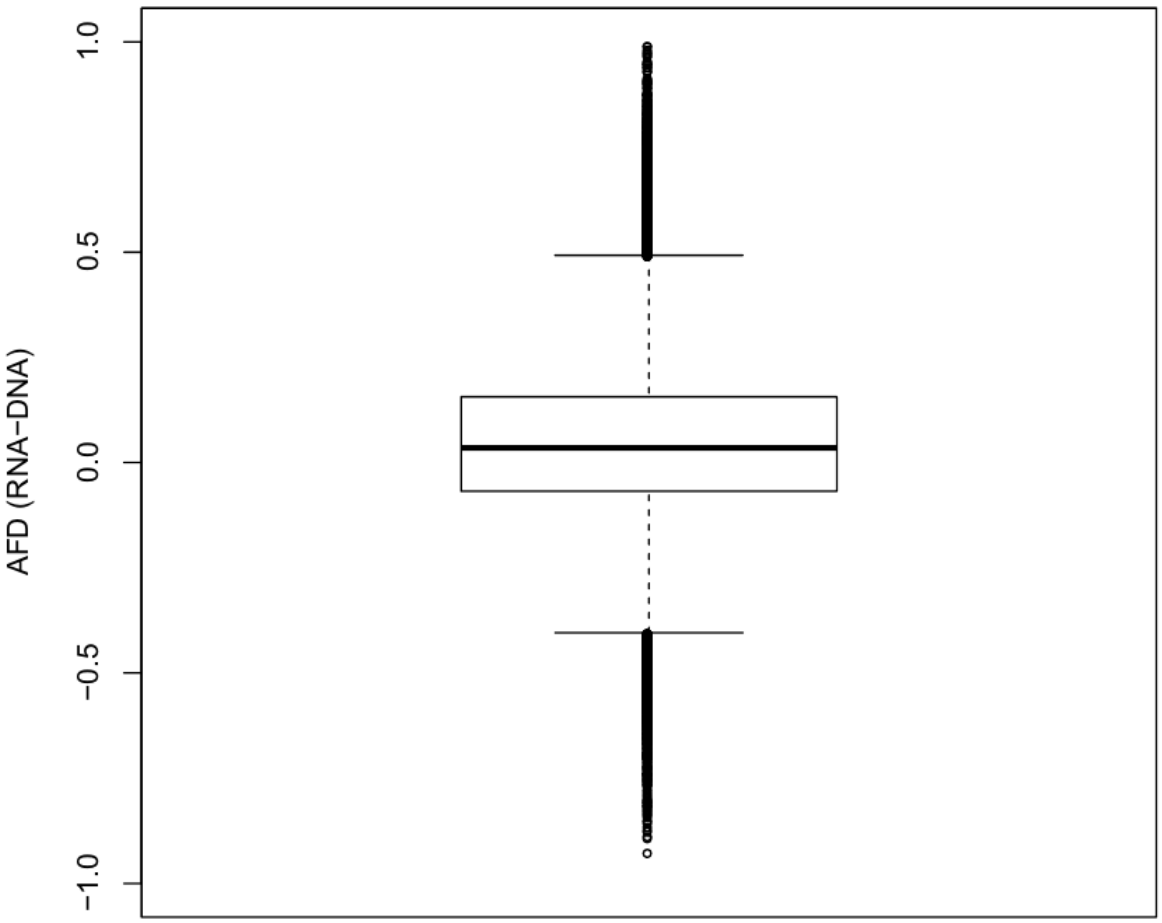

Fig. S2. The distribution of AFD (RNA-VAF minus DNA-VAF) across five tumor types

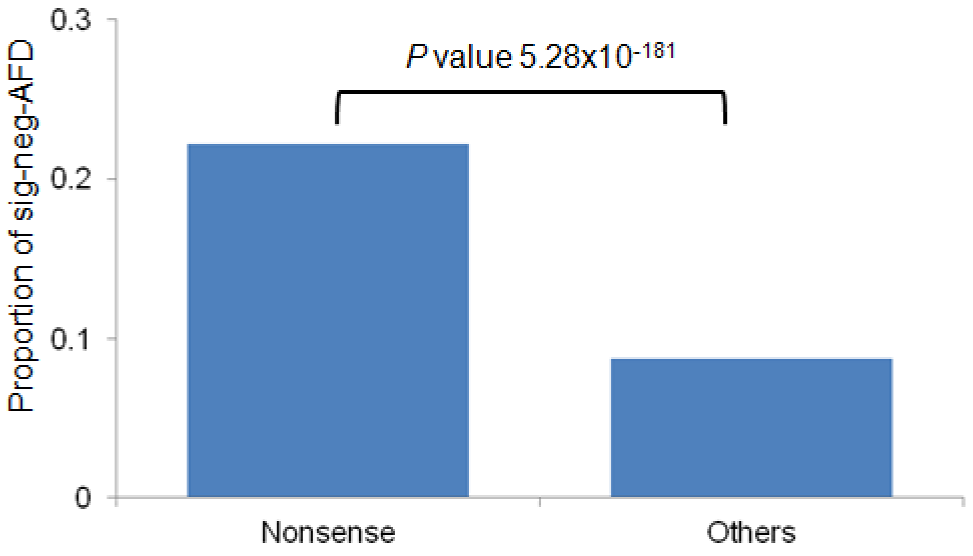

Fig. S3. The proportion of sig-neg-AFD mutant transcripts for the nonsense mutations and other somatic mutation classes

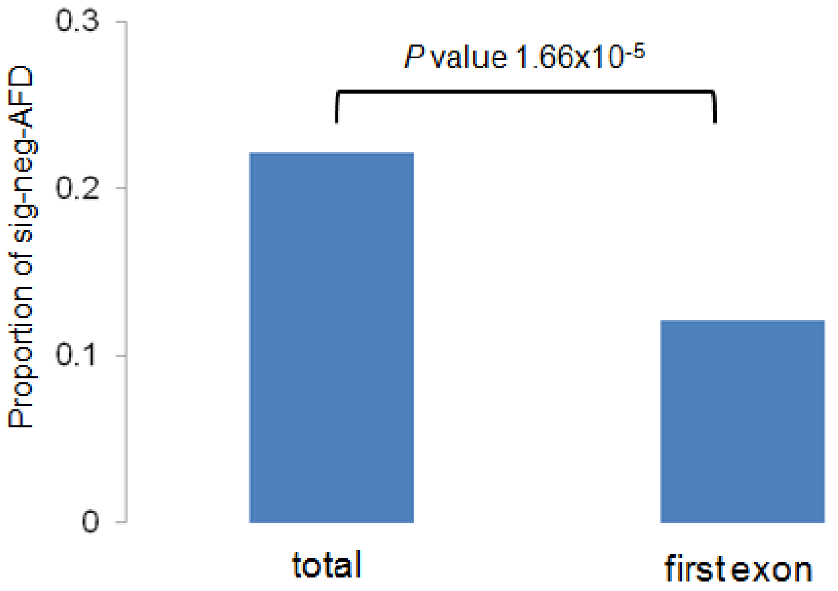

Fig. S4. The proportion of the sig-neg-AFDs in nonsense mutations at the first exon

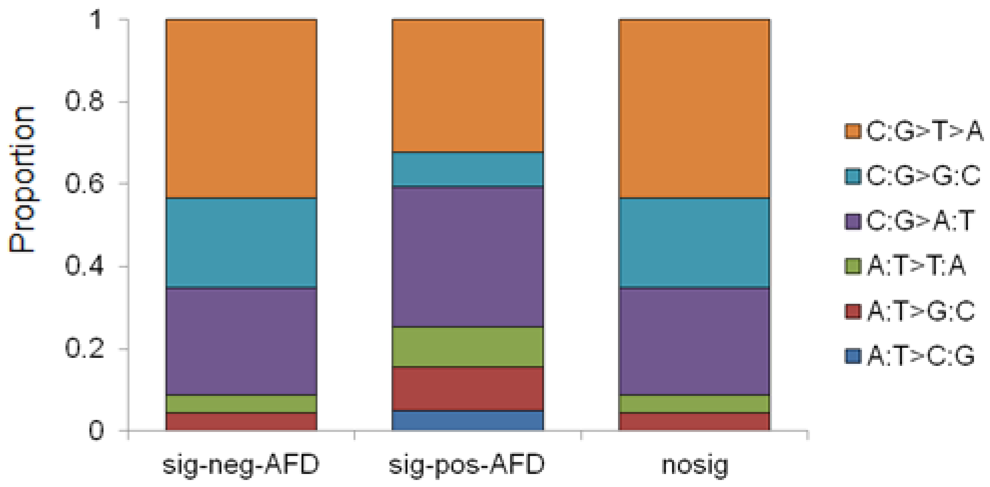

Fig. S5. The mutation spectra was observed with respect to the mutation abundance of sig-pos-AFD, sig-neg-AFD, and no-sig in splice site mutation

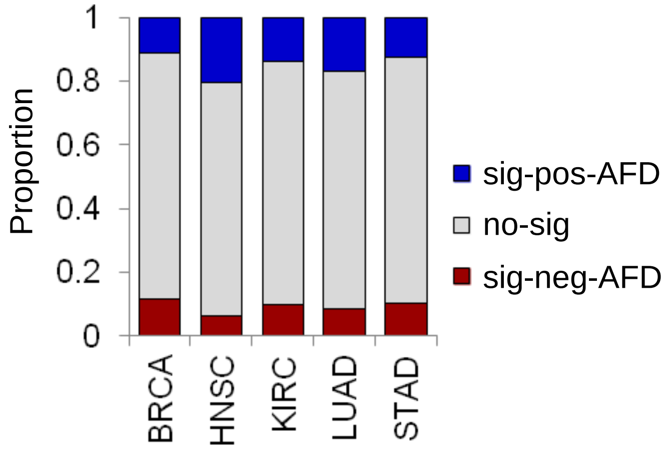

Fig. S6. The proportional barplot for sig-pos-AFD, sig-neg-AFD, and no-sig at missense mutation.

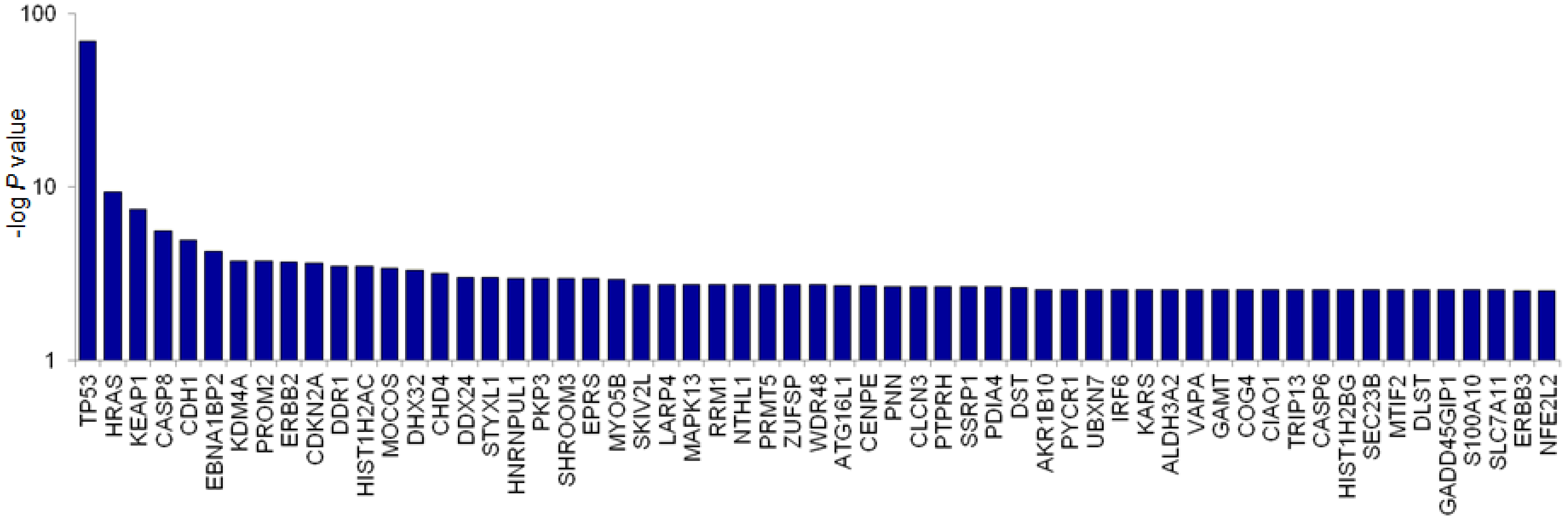

Fig. S7. Genes with frequent sig-pos-AFD across all the cases.

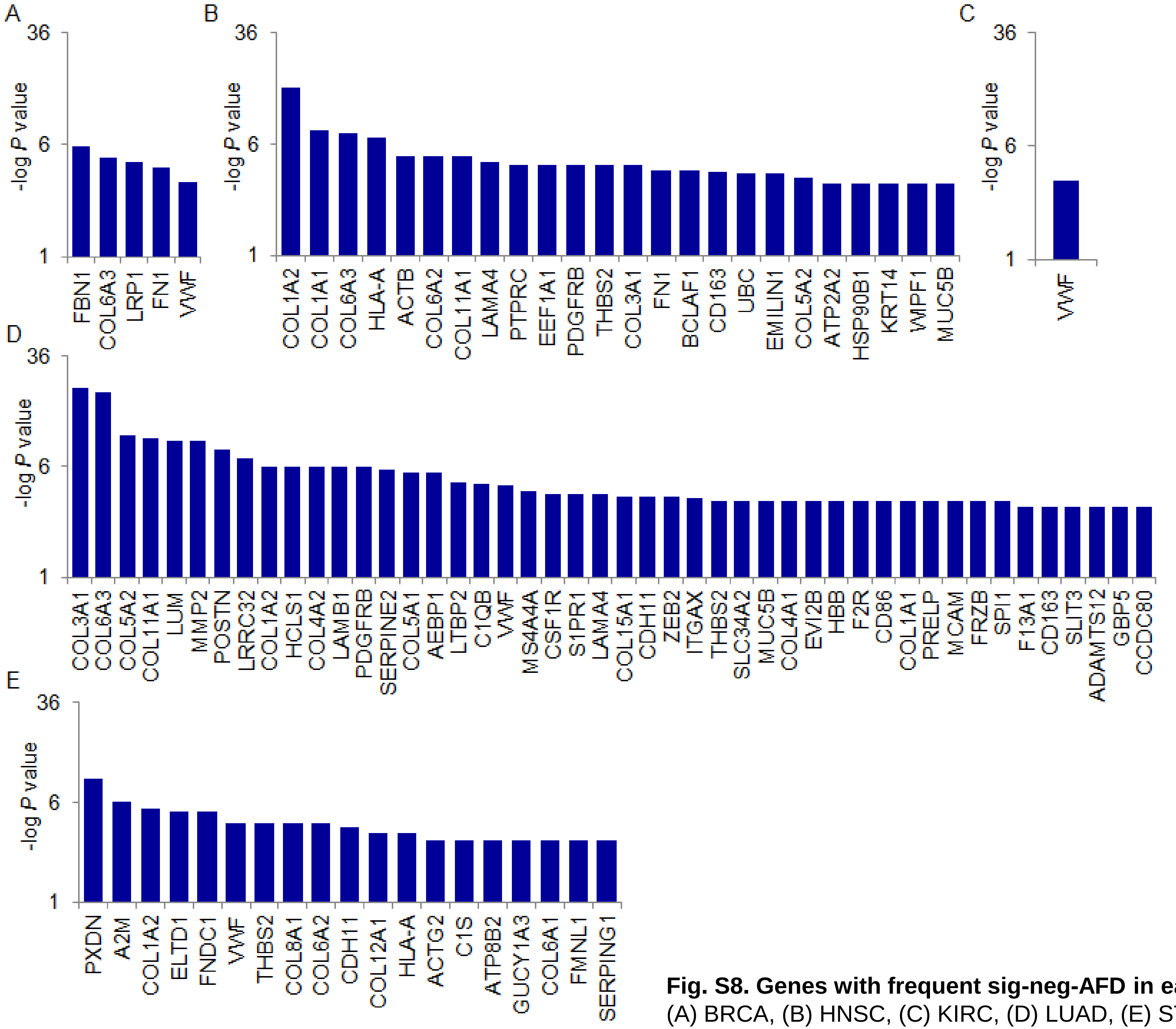

Fig. S8. Genes with frequent sig-neg-AFD in each tumor type. (A) BRCA, (B) HNSC, (C) KIRC, (D) LUAD, (E) STAD

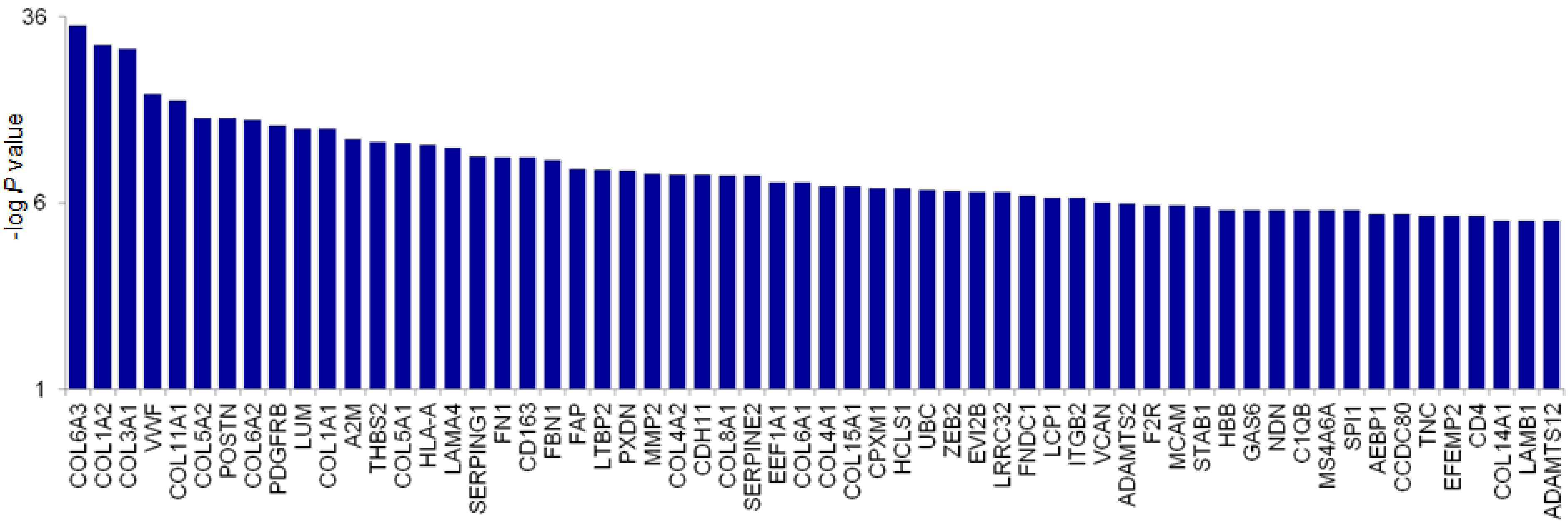

Fig. S9. Genes with frequent sig-neg-AFD across all the cases.

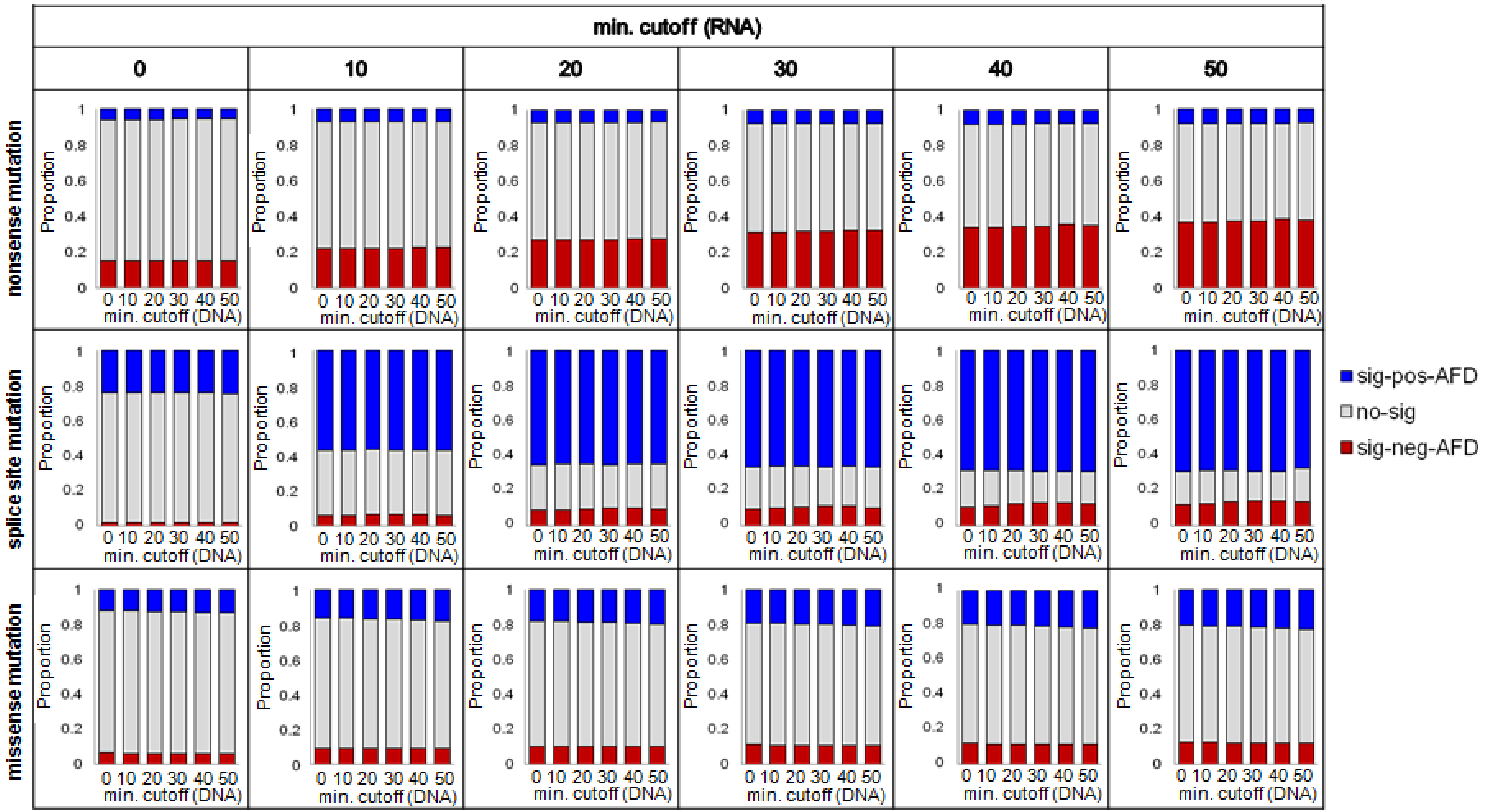

Fig. S10. Proportion of sig-pos-AFD, no-sig, and sig-neg-AFD according to change of minimum cutoff for sequencing coverage depth
